# Sports injuries in patients over 70 years of age. Survey in a regional hospital in an Alpine tourist region: DKOU science slam 2024

**DOI:** 10.1007/s00132-025-04635-6

**Published:** 2025-03-08

**Authors:** Josefina Engelmann, Sabrina Weber, Michel Conti, Henrik Teuber, Hans-Peter Simmen, Samuel Haupt

**Affiliations:** 1 Department of Surgery, Spital Oberengadin, 7503 Samedan, Switzerland; 2 Universitätsspital Zürich, Rämsistr 100, 8090 Zürich, Switzerland

## Infobox Science meets Humor

That is the principle of a science slam. At this short presentation competition researchers present their topics in an entertaining way. The audience decides who best succeeds in bringing entertainment and knowledge into harmony.

The Science Slam is organized as part of the “Deutscher Kongress für Orthopädie und Unfallchirurgie“ (DKOU) every year by the “Young Forum” O and U. The winning team is selected by the audience and a jury according to the three criteria scientific nature, fun factor and volume of applause. The best performance will be awarded the DGOU slam scholarship of the DGOU in the amount of 1000 euros. Springer Medizin Verlag supports the Science Slam with an annual subscription to e.Med Orthopaedics & Trauma Surgery and three annual subscriptions to e.Medpedia.

In 2024, the Science Slam was already in its 7th round, moderated by Dr. Marie Samland. The “slammers” have summarized all four presentations once again in the form of a written article for you.

Would you like to see the format for yourself? Take a look at the Science Slam review of the DKOU 2024: https://jf-ou.de/projekte/science-slam.

## Purpose

Life expectancy in western countries is steadily rising. For example, data from the Swiss Federal Statistical Office show that the life expectancy of patients aged 65 years and over steadily increased from 1876 to 2016 [1]; however, healthy life expectancy is also increasing. This means that there are more and more healthy older trauma patients [2]. This population is still interested in doing sports. According to the data in the literature and official statistics, more sports injuries are observed in hospitals [3], especially in a heavily frequented Alpine tourist region. Alpine regions are particularly popular with athletes as there is a wide range of sports on offer in both winter and summer [4, 5].

Up to now in the literature there is a gap of knowledge for injuries of the 70+ years patient group and especially according to injury patterns due to sports injuries. The current literature provides only very sparse information on this patient group [2, 6, 7]. Even the German trauma registry does not contain any data on sport-related injuries in patients over 70 years of age [8].

Injuries in the age group 70+ years are increasing [9] along with the number of polytraumatized patients [10]. There are data on injury patterns in older patients after falls [9]. Up to now there are no available data on the injury patterns of this age group for sports-related injuries.

Due to the changes of bone density and other ageing processes the injuries in older people may differ from the injuries registered in younger patients [11]. In addition, the ageing process may prolong healing times and the physiological differences in older patients can significantly alter rehabilitation care after an accident [9, 11].

The aim of this study was to assess injury patterns of sport-related injuries for patients aged 70 years and older. We suspect that in winter most injuries occur during skiing rather than snowboarding. We further hypothesize that mild traumatic brain injuries and knee injuries are the most common diagnoses. In summer we expect more accidents with e‑bikes with clavicle fractures and minor traumatic brain injuries being the consequences.

## Methods

### Design.

A single-center retrospective descriptive study was conducted.

### Patient selection criteria.

Patients over 70 years of age who had suffered an injury doing sports from 1 January 2023 to 31 December 2023 were included in the study. Exclusion criteria were accidents not related to sporting activities and refusal for use of personal patient data.

The patients gave consent by a general consent form, by e‑mail or by telephone enquiry. The study was approved by the regional ethics committee. (BASEC No. 2023-01140). It follows the Strengthening the Reporting of Observational Studies in Epidemiology (STROBE) guidelines for reporting observational studies [12].

### Outcome measures.

Data were collected retrospectively from our hospital database (KISIM Cistec AG, Zurich, Switzerland). The basic data included age, date of birth, gender and origin of the patients. The following epidemiological data were collected: date of the accident, injury pattern, diagnosis(es), number of secondary diagnoses, treatment, inpatient or outpatient care, duration of inpatient stay, injury severity score (ISS) and follow-up treatment. The initial research contact took place after the first visit in our trauma bay. Follow-up examinations, especially for outpatients, often took place close to home at different hospitals. The reports of the follow-up examinations of patients receiving further treatment in another hospital were sent to us directly or upon request. For other patients, enquiries were sent by e‑mail or telephone contact directly to obtain follow-up information. This information was a subjective assessment of pain intensity, return to sport and the need for physiotherapy.

Frequencies and percentages for dichotomous and categorical variables were calculated. Mean and standard deviation (SD) were used for normally distributed continuous data. Range was specified for easier data interpretation. Categorical data analysis was performed using Pearsonʼs χ^2^-test. A *p* < 0.05 was considered significant. The analysis was performed with R version 4.4.2 (R Core Team, Ross Ihaka, Robert Gentleman, August 1993) for macOS.

## Results

A total of 158 patients were included: 55 women and 103 men. The average age was 77 years (range: 70–97 years). The average length of hospital stay was 2 days (range: 1–14 days). The most common diagnosis overall was an injury to the head (most of the cases: mild traumatic brain injury, *n* = 28) and was the most common reason for hospitalization. These patients were treated conservatively (Table 2). On average the patients had a minimum of 1 secondary diagnosis not related to the injury (range: 1–8).

**Fig. 1:**
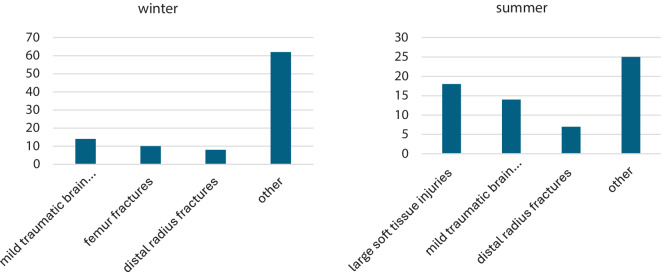
Block diagrams showing the most common injuries (in absolute numbers) recorded divided into summer and winter

The accident mechanisms were different in winter and summer as well as the injury patterns. In winter, mild traumatic brain injury was also the most common reason for emergency consultation. In summer, skin and soft tissue injuries were the most common diagnoses. The second and third most common diagnoses in winter were femoral fractures (*n* = 10) and distal radius fractures (*n* = 8). The most frequent diagnoses in summer were extensive soft tissue injuries (*n* = 18), minor traumatic brain injuries/concussion (*n* = 14) and distal radius fractures (*n* = 7). Other common diagnoses were “other”, such as clavicle fractures, malleolar fractures or contusions (Figure 1).

Tables 1 and 2 show the number of inpatient and outpatient treatments as well as surgical and conservative treatments, broken down into the winter and summer seasons. A total of 65 patients were inpatients and 93 outpatients, 44 patients were treated surgically and 114 conservatively and 4 patients were transferred directly from our emergency department to a central hospital. The reasons for transfers were serious injuries in one patient (average injury severity score ISS of 21 ± 5), incomplete burst fracture of the lumbar spine and in another patient a rotationally unstable pelvic ring fracture with symphysis rupture, a multiple injury with a narrow subdural hematoma on the right frontal (4 mm), a Le Fort fracture type II, including the left orbital floor, a proximal left humeral fracture, bleeding in liver segment VI, a hematoma on the left flank of the abdominal wall and serial rib fractures on the left and right ribs 3–7. The reason for further transfers were a traumatic knee dislocation and an intracranial hemorrhage.

**Table 1:** Numbers of outpatient and inpatient treatment broken down into winter and summer (*p* = 0.55)

	Winter (*n*)	Summer (*n*)	Total (*n*)
Inpatient treatment	41	24	65
Outpatient treatment	53	40	93
Total	94	64	158

**Table 2:** Numbers of surgical and conservative treatment broken down into winter and summer (*p* = 0.14)

	Winter (*n*)	Summer (*n*)	Total (*n*)
Surgical	31	13	44
Conservative	64	50	114
Total	189	127	316

The average ISS of all patients was 6 ± 6. Polytraumatized patients (ISS > 16) had an average ISS of 22 + 6.

Table 3 shows the origins of the treated patients.

**Table 3:** Number of local (Engadin), Swiss and foreign tourists

Local (*n*)	Swiss (*n*)	Foreign (*n*)
30	75	53

The most frequent causes of accidents in winter were skiing falls (*n* = 27, around 30% of collisions) and cross-country skiing falls (*n* = 17). Snowboarding is not very popular in the 70+ years age group. No patient was admitted to hospital after a fall while snowboarding.

In summer, hiking accidents (*n* = 35) were the most common diagnosis. Bicycle or mountain bike (e-bike) falls were less frequent.

## Discussion

The most frequent accident mechanisms were 28 skiing falls and 17 cross-country skiing falls in the winter months (November–April), 25 hiking accidents and 15 mountain bike (e-bike) accidents in the summer months (April–November). The use of e‑bikes is very popular for older people with many more e‑bikes sold since 2010. The age itself seems to carry a greater risk than the type of bicycle (normal bike vs. e‑bike) [13].

Data from the German Trauma Registry show similar events in cycling accidents. For example, data from 2010 to 2019 show that the most common injury in serious cycling accidents is head injuries [5, 14]. Injuries to the head are also the most common when using e‑bikes [13, 15].

In winter there are more people in Engadin than in summer [16, 17]. The risk of injury during sport is significantly higher for winter sports than for summer activities. The favorite sports of injured people aged 70+ years are skiing and cross-country skiing in winter, while snowboarding is not popular at all. The most common injuries in winter were minor traumatic brain injuries caused by skiing. Most patients wore a helmet. This already suggests that this prevents a more serious injury or minimizes the risk [18–20]. Surprisingly, internal knee lesions were found less frequently in older patients compared to the literature on younger patients [21], probably also because we used magnetic resonance imaging (MRI) for diagnostics in older people more restrictively.

As expected, accidents resulting in minor traumatic brain injuries involving e‑bikes are common in summer; however, in summer most patients presented due to skin and soft tissue injuries. In summer, hiking on mountain trails and mountain biking (e-bikes) are among the most popular sports. These sport-related injuries are comparable to injuries of young patients doing the same sports [22]. Therefore, we assume even though the patients are much older, the injury mechanism and correlated injuries are comparable with a younger population group. Nevertheless, this effect could also be explained by more careful handling of sport activities by an older population group and therefore less energy is involved in the accidents. An indication for this lower energy involved could be the high rate of only soft tissue injuries in summer sports and the most frequent injury being mild traumatic brain injury.

Treatment of the patient group 70+ years differs from that of the younger generation. Pre-existing conditions, age and previous medication are important for this change in treatment regimens for the older population; however, our patients had an average of only 1 additional diagnosis. In the literature patients aged 70+ years on average have 8–10 diagnoses as chronic diseases like arterial hypertension [23]. Therefore, we assume that sport in older age is correlated to less comorbidities and less medication intake compared to an average population at the age 70+ years.

### Limitations

We based our diagnostic imaging primarily on conventional X‑rays and computed tomography (CT) scans if indicated. It cannot be guaranteed that we received the complete findings.

Furthermore, this is a single-center retrospective descriptive study. Our study may not represent the general population of 70+ years because the cost of living in Engadin and the fees for the skiing resorts are expensive. Tourists who choose Engadin practice various sports and seem to be quite healthy.

A further limitation is that the numbers of hotel overnight stays mentioned in the text may differ greatly from the actual overnight stays. Many visitors in the Engadin own a holiday flat and are therefore not covered in these statistics. Therefore, we did not do any calculations of overnight stays and number of patients injured.

## Conclusion

Geriatric patients staying active, even though these activities may lead to an injury, have less comorbidities compared to an average population group aged 70 years and older. Accidents caused by skiing are more frequent than those caused by snowboarding in the winter months even for a geriatric population. Minor traumatic brain injuries is the most common diagnosis in older patients suffering a sport-related injury.

## Abbreviations

DKOU: Deutscher Kongress für Orthopädie und Unfallchirurgie
